# Outcome of R-CHOP or CHOP Regimen for Germinal Center and Nongerminal Center Subtypes of Diffuse Large B-Cell Lymphoma of Chinese Patients

**DOI:** 10.1100/2012/897178

**Published:** 2012-11-04

**Authors:** Ying Huang, Sheng Ye, Yabing Cao, Zhiming Li, Jiajia Huang, He Huang, Muyan Cai, Rongzhen Luo, Tongyu Lin

**Affiliations:** ^1^State Key Laboratory of Oncology in South China, Sun Yat-sen University Cancer Center, 651 Dongfeng Road East, Guangzhou 510060, China; ^2^Department of Radiation Oncology, Sun Yat-sen University Cancer Center, 651 Dongfeng Road East, Guangzhou 510060, China; ^3^Department of Medical Oncology, The First affiliated Hospital of Sun Yat-sen University, 58 zhongshan Road, Guangzhou 510080, China; ^4^Departments of Oncology, Kiang Wu Hospital, Kiang Wu Road, Macau, China; ^5^Department of Medical Oncology, Sun Yat-sen University Cancer Center, 651 Dongfeng Road East, Guangzhou 510060, China; ^6^Department of Pathology, Sun Yat-sen University Cancer Center, 651 Dongfeng Road East, Guangzhou 510060, China

## Abstract

Diffuse large B-cell lymphoma (DLBCL) can be molecularly subtyped as either germinal center B-cell (GCB) or non-GCB. The role of rituximab(R) in these two groups remains unclear. We studied 204 patients with de novo DLBCL (107 treated with first-line CHOP; 97 treated with first-line R-CHOP), patients being stratified into GCB and non-GCB on the basis of BCL-6, CD10, and MUM1 protein expression. The relationships between clinical characteristics, survival data, and immunophenotype (IHC) were studied. The 5-year overall survival (OS) in the CHOP and R-CHOP groups was 50.4% and 66.6% (*P* = 0.031), respectively. GCB patients had a better 5-year OS than non-GCB patients whether treated with CHOP or not (65.0% versus 40.9%; *P* = 0.011). In contrast, there is no difference in the 5-year OS for the GCB and non-GCB with R-CHOP (76.5% versus 61.3%; *P* = 0.141). In non-GCB subtype, additional rituximab improved survival better than CHOP (61.3% versus 40.9%; *P* = 0.0303). These results indicated that addition of rituximab to standard chemotherapy eliminates the prognostic value of IHC-defined GCB and non-GCB phenotypes in DLBCL by improving the prognostic value of non-GCB subtype of DLBCL.

## 1. Introduction

Diffuse large B-cell lymphoma (DLBCL) is the most common subtype of non-Hodgkin's lymphomas, representing 30% of all newly diagnosed cases and more than 80% of aggressive lymphomas [1]. DLBCL is a heterogeneous disease, and multiple morphologic variants have been recognized within the WHO 2008 classification system. It is likely that advances in molecular biology will allow the current classification to be augmented, with the recognition of newer entities and the homogenization of lymphoma subtypes. Recent studies that have distinguished the cell of origin have provided a prognostic and biologically relevant subclassification of DLBCL. Germinal center B-cell (GCB) and non-GCB subtypes, which were originally characterized by gene expression studies [2], have subsequently been validated at the protein level using IHC, as introduced by Hans et al. [3]. In first-line therapy with conventional CHOP, which has been the standard chemotherapy regimen for DLBCL for more than two decades, patients within the GCB group have a better 5-year survival than patients within the non-GCB subgroup [4]. Therefore, the GCB or non-GCB characteristic can be regarded as a new prognostic factor for DLBCL patients. In patients treated with a combination of rituximab and chemotherapy, the clinical significance of the GCB/ABC subtyping is more controversial [5]. Risk assessment is moving forward within the monoclonal antibody era, and new therapies that are being introduced can significantly alter the relevance of previously recognized prognostic factors by virtue of their mechanism of action [6].

Nearly all the studies of prognostic indicators in DLBCL, including the IPI, have been based on the clinical outcome following treatment with CHOP [7]. Although several factors appear to predict outcome and survival rates for patients undergoing chemotherapy, it may be that these factors are not as efficient in predicting response to biologics such as rituximab [8]. Although the adoption of R-CHOP (rituximab in combination with CHOP) as the new standard of care has led to improved outcomes for DLBCL, patients who fail in first-line therapy remain a difficult challenge. In the era of rituximab, the questions as to whether the prognostic markers for conventional therapy are still valid and whether these markers should still be used to guide treatment choice deserve consideration [9].

In the present study, we explore the outcome of addition of rituximab into CHOP regimen among GCB and non-GCB in our institutes and address whether IHC-defined GC versus non-GC distinction of DLBCL could be used to predict a patient's outcome in response to a combination of rituximab and chemotherapy. 

## 2. Materials and Methods

### 2.1. Patient Selection and Tumor Specimens

We retrospectively studied 204 patients with de novo DLBCL diagnosed at Sun Yat-sen University Cancer Center between 1998 and 2005. All of these patients received either the CHOP (*n* = 107) or R-CHOP (*n* = 97) regimen as first-line chemotherapy. The selection criteria were the availability of paraffin-embedded tumor biopsies for IHC analysis and detailed information on treatment and followup. Patients with T-cell lymphoma or B-cell lymphoma other than DLBCL were excluded. 

Formalin-fixed, paraffin-embedded tumor sections were available in all cases and were reviewed by two pathologists, who confirmed that all cases were de novo DLBCL according to the 2008 WHO lymphoma classification system.

The extent of disease had been determined at first presentation by physical examination, serum lactate dehydrogenase concentration, full blood count, and computed tomography of the chest and abdomen. For each patient, the following characteristics were noted from the medical records: age at diagnosis, sex, Ann Arbor stage at presentation, therapy, achievement of complete remission, occurrence of relapse, and time to death or loss to followup.

### 2.2. Immunohistochemistry Study

Formalin-fixed, paraffin-embedded tumor sections were examined for the expression of CD10, BCL-6, and MUM1. Briefly, slides were deparaffinized by xylene and rehydrated. Tissue sections were antigen-retrieved in Tris EDTA buffer (10 mM/L, pH 8.0) and incubated by heat induction for 20 minutes (CD10 and MUM1) or 40 minutes (BCL-6). The mouse anti-IRF4 antibody (diluted 1 : 400; clone M17, DAKO, Glostrup, Denmark), mouse anti-BCL-6 antibody (diluted 1 : 40; clone M17, DAKO, Glostrup, Denmark), and mouse anti-CD10 antibody (diluted 1 : 50; Zymed) were used. Sections of reactive tonsil and PAS were used as positive and negative controls, respectively.

The proportion of positively stained tumor cells was estimated by two pathologists who had no knowledge of the corresponding clinical data. Disagreements were resolved by reanalysis of the staining. Tumor cells positive for the markers were evaluated semiquantitatively with a cutoff of 30%. All cases were subdivided into GCB or non-GCB subtypes as described by Hans; briefly, the phenotype of GCB was defined as CD10 positive (regardless of the other two markers) or CD10−BCL6+MUM1−. The non-GCB phenotype was defined as CD10−BCL6−MUM1+ or lack of expression of all three markers. 

### 2.3. Treatment Response and Survival Evaluation

The response criterion of non-Hodgkin's lymphomas was applied to determine the occurrence of complete remission (CR), partial remission (PR), stable disease (SD), or progressive disease (PD). Overall survival (OS) was measured from the date of diagnosis until the last followup or death from any cause. Progression-free survival (PFS) was determined as an interval between the date of diagnosis and relapse, or death. 

The relationships between the three markers, the subdivision and OS were assessed by Kaplan-Meier graphs. Fisher's exact test was used to identify significant correlations between variables. The *P* values for these analyses are based on the log-rank test. Cox proportional hazard multivariate analysis was performed to compare the prognostic importance of the different variables. SPSS 12.0 for Windows software was used for all assessments.

## 3. Results

### 3.1. Patient Characteristics

Of the 204 patients, 107 were treated initially with the standard CHOP regimen and 97 were treated with R-CHOP. The clinical data, including IPI, was retrospectively evaluated in all patients. A total of 202 of the 204 patients had all the necessary data available to calculate the IPI; two of 204 patients had no record of serum lactate dehydrogenate concentration. For the CHOP group, 107 DLBCL patients aged 16–84 years (median age, 54 years) were included. The follow-up period ranged from 6 to 167 months (median, 53 months). For the R-CHOP group, 97 DLBCL patients aged 20–83 years (median, 59.0 years) were studied. The follow-up period for the R-CHOP group ranged from 3 to 106 months (median, 56 months). Patient and disease characteristics for both treatment cohorts, including the five clinical parameters that comprise the IPI, are listed in Table 1. Otherwise, the clinical features were not significantly different between the rituximab and control groups. The distribution of IHC-defined GCB and non-GCB phenotypes was also similar between the two groups. The GCB and non-GCB groups were similar with regard to age and sex distribution. Among the 107 patients treated with CHOP, there were 44 in the GCB subgroup and 63 in the non-GCB subgroup; this ratio being consistent with previous reports. In both groups, the distributions of sex, age, stage of disease, and origin of disease were equivalent. However, there were more patients with a low IPI score in the GCB subgroup (36.4% versus 4.7%; *P* = 0.012). Among the 97 patients treated with R-CHOP, there were 33 in the GCB subgroup and 64 in the non-GCB subgroup. There was no difference in the frequency of low IPI scores between the GCB and non-GCB subgroups (23.7% versus 10.3%; *P* = 0.050).

### 3.2. Immunohistochemistry Results

The expression patterns of CD10, BCL-6, and MUM1 among the patients treated with CHOP and R-CHOP are summarized in Tables 2 and 3. Among all 204 patients, the rates of expression of CD10, BCL-6, and MUM1 were 32.3%, 33.4%, and 43.6%, respectively. The expression patterns of the three proteins were similar in the two groups. We successfully grouped all patients into GCB or non-GCB subtypes. In total, there were 97 and 107 patients who showed the GCB and non-GCB profile, respectively. Among the patients who received CHOP, MUM1 expression was seen in 7/46 GCB cases and 41/63 non-GCB cases. There was a significant difference in the percentage of cases exhibiting MUM1 positivity between the GCB and non-GCB subgroups (*P* < 0.0001). Among the patients who received R-CHOP, MUM1 expression was seen in 2/33 GCB cases and 26/64 non-GCB cases (*P* = 0.005), which made it the most important marker of the three. 

### 3.3. Survival Analysis and Response to Treatment

To evaluate the prognostic efficacy of the three factors, we performed survival analyses based on the individual markers alone and in combination. First, we evaluated the role of additional rituximab in DLBCL patients. We compared patient outcomes between the CHOP group and the R-CHOP group. A significant difference in outcome was observed between the two groups. According to the Kaplan-Meier estimates, the 5-year OS rates were 54.0% in the CHOP group and 66.6% in the R-CHOP group (*P* = 0.031). Similarly, the 5-year progression-free survival (PFS) was 64.9% and 48.9% for the R-CHOP and CHOP groups, respectively (*P* = 0.007; data not shown). Therefore, we confirmed that the addition of R to standard chemotherapy showed a proof-of-survival benefit in the present study.

### 3.4. Subclassification on the Basis of the Cell of Origin Is Predictive of Survival in Patients with DLBCL Who Were Treated with CHOP but Not with R-CHOP

**Figure 1:**
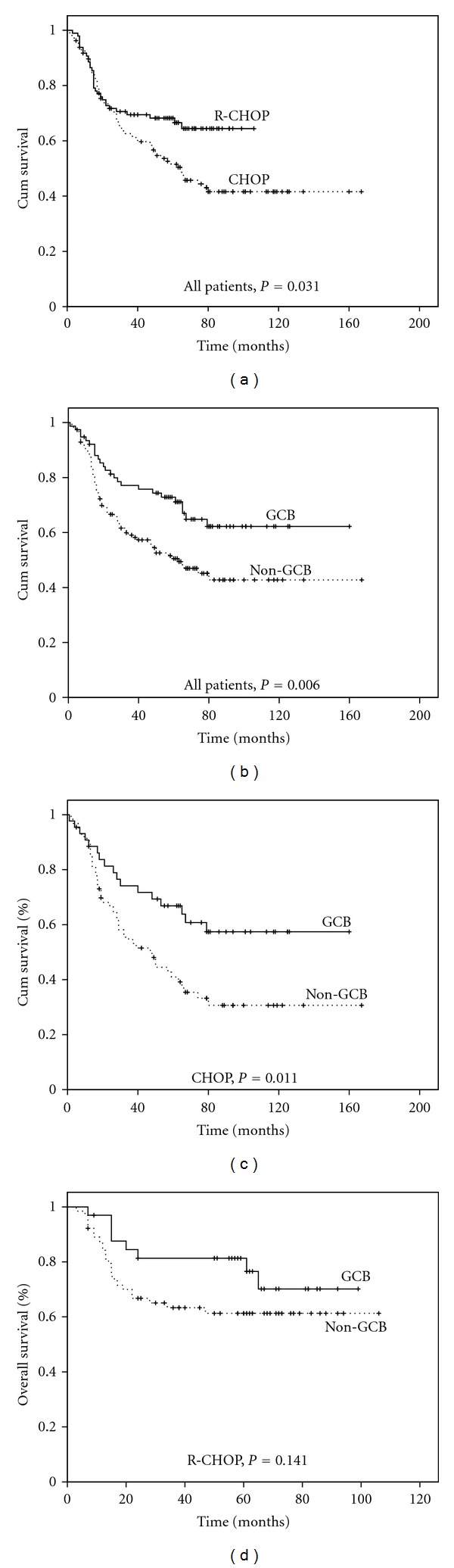
Kaplan-Meier survival curves for all DLBCL patients treated with CHOP or R-CHOP. (a) OS of all DLBCL patients treat with CHOP (*n* = 107) or R-CHOP (*n* = 97). (b) OS according to immunohistochemically defined GCB (*n* = 77) versus non-GCB (*n* = 127) distinction for all patients. (c) OS according to immunohistochemically defined GCB (*n* = 44) versus non-GCB (*n* = 63) distinction for patients treated with CHOP. (d) OS according to immunohistochemically defined GCB (*n* = 33) versus non-GCB (*n* = 64) distinction for patients treated with R-CHOP.

We demonstrated that subgrouping determined by the cell of origin on the basis of IHC successfully predicted the prognosis of DLBCL patients treated with the standard CHOP regimen. Clinical outcomes defined immunohistologically as GCB versus non-GCB subtypes are shown in Figures 1(a) and 1(b). The survival rate in the CHOP group was significantly better for the GCB subtype than for the non-GCB subtype (Figure 1(c)). Among these patients, the 5-year survival was 65.0% in the GCB subgroup and 40.9% in the non-GCB subgroup (*P* = 0.011). CR rates in the GCB and non-GCB subgroups were 72.7% and 49.2%, respectively (*P* = 0.012). However, among the patients treated with R-CHOP, no significant difference was found in the 5-year OS between the GCB and non-GCB subgroups (76.5% versus 61.3%; *P* = 0.141). The 5-year PFS was 73.0% and 61.0%, respectively (*P* = 0.146); the CR rates were 72.7% and 46.9%, respectively (*P* = 0.013) (Figure 1(d)). 

### 3.5. Survival in GCB or Non-GCB Patients Treated with CHOP or R-CHOP

**Figure 2:**
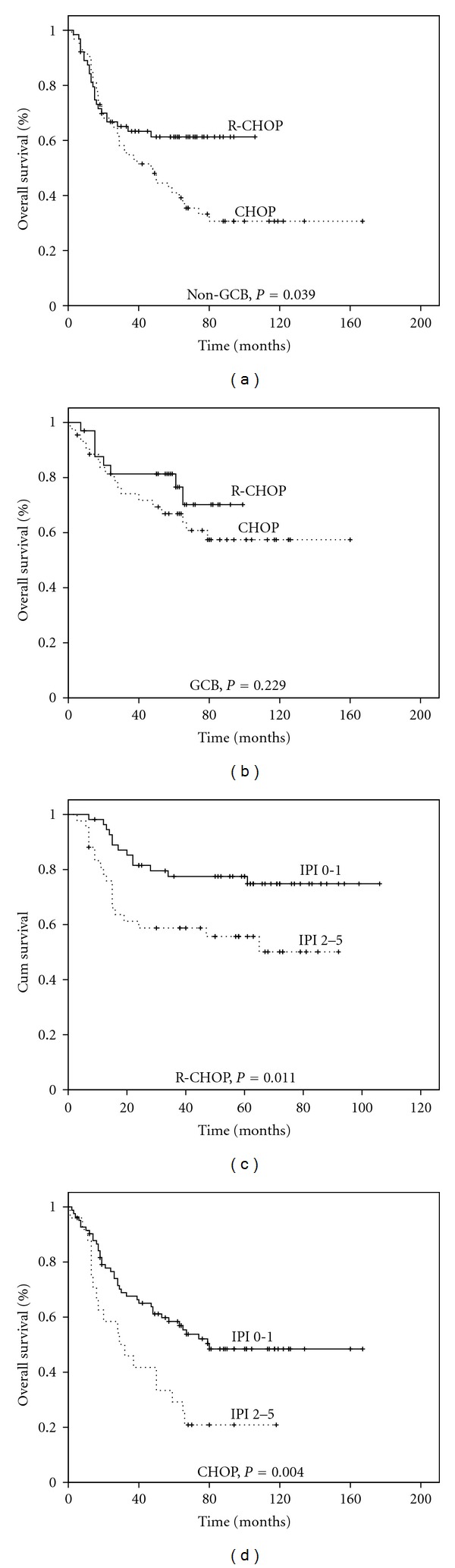
Kaplan-Meier survival curves for GCB or non-GCB subgroup DLBCL patients treated with CHOP or R-CHOP. (a) OS of GCB subgroup treat with CHOP (*n* = 44) or R-CHOP (*n* = 33) (b) OS of GCB subgroup treat with CHOP (*n* = 63) or R-CHOP (*n* = 64). ((c)-(d)) OS according to IPI for patients treated with CHOP (IPI 0-1, *n* = 82 versus IPI 2–5, *n* = 25) or R-CHOP (IPI 0-1, *n* = 55 versus IPI 2–5, *n* = 42).

To study the impact of rituximab on the predictive value of subclassification on the basis of the cell of origin, we examined the survival outcomes according to treatment in the GCB or non-GCB subgroups, as defined by IHC stains. Among the GCB subgroup, no significant difference was found in the 5-year OS of patients treated with CHOP and R-CHOP which were 66.5% and 76.5% (*P* = 0.229) (Figure 2(b)). The similar results were noticed in 5-year PFS (63.0% versus 73.0%, *P* = 0.262, data not shown). However, there were great significant differences in the 5-year OS of non-GCB patients treated with CHOP or R-CHOP (61.2% versus 40.9%, *P* = 0.039) (Figure 2(a)). The similar results were noticed in 5-year PFS (33.7% VS 61.0%, *P* = 0.005, data not shown).

### 3.6. IPI Was the Prognostic Fact for Both CHOP and R-CHOP Groups

We also explored the prognostic significance of the IPI. We used IPI scoring system, instead of individual included factors in IPI, and subgrouped the patient into low risk (IPI score 0 and 1) and high risk (IPI score more then 2). In the CHOP group 34, 47, 21, 5, and 0 patients had an IPI score 0 to 4, respectively. The 5-year OS was 66.5%, 45.8%, and 30.0% for IPI scores 0, 1, and 2 (*P* = 0.018). In the R-CHOP group, 20, 35, 32, 8, and 2 patients had an IPI score 0 to 4. The 5-year OS was 92.8%, 64.2%, 54.3%, and 31.2% for IPI scores 0, 1, 2, and 3 (*P* = 0.028). Next, patients with low risk and high risk were evaluated for survival in both groups. In the CHOP group, the 5-year survivals for the low-risk and high-risk groups were 56.9% and 29.2%, respectively (*P* = 0.004). In the R-CHOP group, the 5-year survivals for the low-risk and high-risk groups were 74.8% and 50.0%, respectively (*P* = 0.011). So IPI was a good predictor of prognosis in these patients (Figures 2(c) and 2(d)).

By the Cox proportional hazards regression model, in the CHOP group, the IHC-defined GCB phenotypes and clinical characters such as males, young patients, and early stages were associated with a significantly favorable survival rate, independently of other IPI parameters. Whereas these factors were not independently significant prognostic factors in the R-CHOP group (Table 3).

## 4. Discussion

DLBCL is a heterogeneous disease, as the microarray analysis showed that patients with DLBCL expressing a gene expression profile (GEP) of germinal center B cells (GCB) have a longer survival than patients of activated B cells (ABC) [10]. Since the clinical utility is limited by high cost of microarray analysis, many algorithms were introduced to stratify DLBCL based on the IHC expression profile of CD10, BCL-6, and MUM1 [3, 11]. 

The aim of our study was to identify whether cell-of-origin distinction has prognostic impact on DLBCL patients treated with combination of rituximab and chemotherapy. We confirmed that patients with DLBCL treated with CHOP alone can be stratified into low-risk and high-risk subgroups by cell of origin. In line with previous Western clinical studies [12, 13], our data demonstrate that addition of rituximab to chemotherapy improves the outcome of DLBCL patients of all ages and risk groups in China. We also provided a longer follow-up data then others, the maximum and median follow-up times about 12 years and 51 months, respectively. The 5-year OS for GCB and non-GCB patients treated with CHOP in our series was 65.0% and 40.9%, respectively (*P* = 0.011). We confirmed that R-CHOP improved the prognostic value of the IHC-defined non-GCB subtype of DLBCL. Our data suggested that R-CHOP improved the outcome of non-GCB subgroup (61.3% versus 40.9%; *P* = 0.0303), but not in the GCB subgroup (76.5% versus 61.3%; *P* = 0.141). Taken together, these data seem to suggest that rituximab eliminates the prognostic value of IHC-defined GC and non-GC phenotypes in DLBCL, as also shown in other studies [14, 15].

Several studies have suggested that prognostic factors for patients with non-Hodgkin's lymphoma have changed for those treated with rituximab, and patient characteristics may differ between those treated with chemotherapy or rituximab. Czuczman et al. analyzed the characteristics of 166 patients with non-Hodgkin's lymphoma (including 130 patients with follicular lymphoma) in a phase III trial of rituximab; results showed that the Follicular Lymphoma International Prognostic Index did not correlate consistently with the response to rituximab or the response duration [16]. In our series, for patients treated with CHOP, the OS in the GCB subgroup was significantly better than that in the non-GCB subgroup. However, such a difference did not exist in patients treated with R-CHOP, which suggests that the expression of germinal center markers does not correlate with a more favorable outcome in the rituximab era.

The mechanism is unknown but a chemosensitizing effect of the antibody was suggested in previous study [17]. Many clinical studies have demonstrated that the poor outcome of ABC-like DLBCL might relate to the constitutive activation of the nuclear factor kappa *β* pathway [18, 19]. Lymphoma cell culture studies also showed that rituximab may suppress the constitutively active NF-*κ*B pathway in the non-GC-type DLBCL via significantly upregulating RKIP expression, resulting in decreased activity of the NF-*κ*B pathway and diminishing NF-*κ*B DNA-binding activity [20] and further leading to the enhanced sensitivity of chemotherapy. 

With the gain insight of these molecular characteristics, many studies are ongoing to explore new treatments among the poor prognosis of IHC-defined non-GCB subtype DLBCL patients (NCT00931918, NCT00736450). These studies will assess the effectiveness of R-CHOP in combination with bortezomib and antisense BCL-2 antibody in previously untreated non-GCB DLBCL patients.

Although IHC algorithms and possibly other methods are promising tools for predicting cell of origin of DLBCL as part of routine pathologic diagnosis, improvements of these techniques are needed. In line with other studies, the main problem in our study was the poor methodological standardization due to nonuniform technology. Prospective studies would obtain more reliable information than retrospective studies. Our results demonstrate that prognostic factors based on the cell of origin correlate with significantly different OS rates in patients treated with CHOP; however, no difference is observed in the survival rates of patients treated with R-CHOP, which indicates that rituximab may improve the poor prognosis of patients with high-risk DLBCL. To properly reevaluate the existing prognostic factors, prospective studies using uniform technology and standardized methodology will be required.

## Figures and Tables

**Table 1:** Characteristics of DLBCL patients treated with CHOP or R-CHOP.

Characteristics	Total			CHOP			RCHOP		
	CHOP	RCHOP	*P *	GCB	Non-GCB	*P *	GCB	Non-GCB	*P *
Sex									
Male	65	60	0.871	26	39	0.769	19	41	0.633
Female	42	37		18	24		14	23	
Age									
Young	77	58	0.066	32	45	0.833	21	37	0.579
Elder	30	39		12	18		12	27	
LDH									
Normal	66	56	0.565	28	38	0.728	23	33	0.086
High	41	41		16	25		10	31	
Stage									
I	46	18		20	26		9	9	
II	36	39		16	20		14	25	
III	18	26		6	12		4	22	
IV	7	14		2	5		6	8	
Site									
Nodal	82	79	0.400	34	48	0.896	26	53	0.629
Extra nodal	25	18		10	15		7	11	
IPI									
Normal	82	55	0.002	39	43	0.014	23	32	0.063
High	25	42		5	20		10	32	

**Table 2:** Results of the different immunohistochemistry staining in relation to overall survival in CHOP and R-CHOP subgroups.

Characteristics	Expression	CHOP (*n* = 107)			R-CHOP (*n* = 97)		
		*N *	OS	*P *	*N *	OS	*P *
CD10	Negative	71	47.9	0.297	67	61.5	0.139
	Positive	36	54.8		30	77.6	

BCL-6	Negative	72	52.5	0.350	64	67.8	0.858
	Positive	35	49.2		33	64.9	

MUM1	Negative	59	60.0	0.252	56	67.0	0.780
	Positive	48	40.2		41	65.8	

Cell origin	GCB	44	65.0	0.011	33	76.5	0.141
	Non-GCB	63	40.9		64	61.3	

**Table 3:** Cox proportional hazard regression analysis for CHOP and R-CHOP groups.

	CHOP				R-CHOP			
	RR	95% CI		*P *	RR	95% CI		*P *
		Lower	Upper			Lower	Upper	
OS								
GCB versus non-GCB	2.125	1.196	3.776	0.010	1.712	0.757	3.872	0.197
Sex (male versus female)	2.057	1.192	3.550	0.010	0.550	0.240	1.260	0.158
Age (young versus elder)	3.350	1.911	5.871	0.000	1.972	0.853	4.562	0.112
Stage (early versus advance)	1.932	1.066	3.502	0.030	0.559	0.173	2.068	0.418
PFS								
IPI (0-1 versus 2–5)	1.156	0.438	3.051	0.770	2.408	1.187	4.883	0.015
GCB versus non-GCB	2.463	1.385	4.382	0.003	1.664	0.739	3.747	0.219
Sex (male versus female)	1.869	1.102	3.170	0.020	0.562	0.247	1.276	0.168
Age (young versus elder)	3.464	1.994	6.017	0.000	2.473	1.223	5.002	0.012

RR indicates relative risk; CI: confidence interval; OS: overall survival; PFS: disease-free survival.
